# Between bullets and outbreaks: epidemic control in Sudan’s forgotten war

**DOI:** 10.1186/s13031-026-00763-8

**Published:** 2026-03-23

**Authors:** Salma Ibrahim Mohammed Adam, Rawa Badri, Iyas Dawood, Safa Abdalrhim, Alaa Elsaeed, Mohamed Idries, Lina Hemmeda

**Affiliations:** 1 End the Neglect Initiative, Research and Development, Khartoum, Sudan; 2 Faculty of Medicine, University of Khartoum, Khartoum, Sudan; 3 Mycetoma Research Centre, University of Khartoum, Khartoum, Sudan; 4 Faculty of Medicine, Omdurman Islamic University, Khartoum, Sudan; 5 Faculty of Medicine, National Ribat University, Khartoum, Sudan

**Keywords:** Cholera, Conjunctivitis, Polio, Measles, Malaria, Dengue, War, Epidemic, Sudan

## Abstract

Two years into Sudan’s prolonged war, the collapse of health systems has fueled multiple concurrent epidemics, triggering a complex humanitarian emergency with regional spillover risks. Over 30 million people need aid, more than 11 million displaced internally, and cross-border movement has exacerbated the spread of vaccine-preventable diseases, including Cholera, Measles, and circulating vaccine-derived Poliovirus type 2 (cVDPV2). This review analyzes emerging epidemiological patterns and operational barriers to epidemic control in the context of armed conflict, displacement, and infrastructure collapse. It concludes with targeted recommendations for conflict-sensitive public health interventions.

## Introduction

Two years since the eruption of war in Sudan. The conflict between the two forces, the Sudan Armed Forces (SAF) and the Rapid Support Forces (RSF), has escalated into a prolonged, multi-sided war that has devastated essential services, like healthcare. Humanitarian agencies report that more than 30 million Sudanese urgently need aid. The conflict has triggered the largest internal displacement crisis globally, with over 11 million internally displaced persons (IDPs) and more than 3.9 million refugees fleeing into neighboring countries [1, 2].

At least 114 health facilities have been damaged or forced to close, and attacks on healthcare infrastructure and personnel have exceeded 550 documented incidents since the conflict began [3]. The recent lean season has further complicated the situation; thousands of people have been affected by heavy rains and flooding across 15 states in Sudan [2]. Acute malnutrition rose during these seasons as roads and bridges were destroyed, and access to food became limited [1]. In this vacuum, cholera, measles, conjunctivitis, and poliovirus outbreaks have either emerged or worsened. The surveillance and early warning systems have collapsed in many affected areas, further limiting outbreak detection and response [4]. 

In late 2024, famine was confirmed in parts of North Darfur and the Nuba Mountains, with projections indicating further spread in the coming months [1, 4]. In overcrowded displacement sites such as Zamzam and Abu Shouk, the combination of unsafe water, poor sanitation, and insufficient shelter has created ideal conditions for epidemic transmission. Disease outbreaks in these camps are now worsening pre-existing vulnerabilities.

Efforts to control the spread of epidemics remain constrained by many barriers. Cross-border aid routes from Chad and South Sudan are inconsistently permitted, and humanitarian staff face various restrictions, with only 40% of visa requests by INGOs approved in the six months leading to March 2025^1^. Despite the scale of need, international funding has lagged. By April 2025, Sudan’s Humanitarian Response Plan had received just 10% of the required funds, and over 80% of US-funded health projects had been discontinued [1, 4].

This article discusses how structural violence and displacement fuel the risk and spread of infectious disease outbreaks. We highlight the challenges that face control of the emerging epidemics and drivers of vulnerability and provide recommendations to help control these outbreaks.

### Epidemiological overview

The current humanitarian crisis has significantly altered the epidemiological landscape in Sudan, with resurgent infectious diseases reflecting the collapse of basic public health infrastructure. While cholera, keratoconjunctivitis, and polio are not new to the region, their resurgence underlines the broader systemic breakdown. It provides an alarming signal of how protracted conflict can reverse decades of disease control. Moreover, the rising incidences of measles, dengue, and malaria have compounded the public health challenges, amplifying the vulnerabilities of already overstretched populations.

### Cholera outbreaks

The cholera outbreaks in 2024–2025 are not merely the result of seasonal trends or rainfall but are intricately tied to the destruction of water and sanitation infrastructure due to the conflict. White Nile and Kosti, once relatively stable, have become epicenters with alarming rates, over 2,700 cases and nearly 100 deaths in White Nile, and 58 deaths among 1,300 cases in Kosti in just three days [5].

This sharp increase in incidence and lethality is likely due to delays in case detection and weakened health infrastructure. According to the WHO Multi-Country Cholera Situation Report, Sudan is among the top five countries contributing to the global cholera burden in early 2025 [6]. The lack of access to oral rehydration therapy and intravenous fluids, coupled with delayed referral systems, has contributed to higher-than-expected case fatality rates.

In contrast, countries with faster mobilization and coordinated vaccine distribution, such as Malawi in 2022, contained outbreaks with far fewer fatalities. This comparison underscores the critical impact of governance and logistics on public health outcomes [7].

### Keratoconjunctivitis

Keratoconjunctivitis, while less frequently reported in formal surveillance, has shown increasing prevalence in displacement shelters and conflict zones. Poor hygiene conditions, dust exposure, and shared water use contribute to transmission, particularly in vulnerable groups such as children and the elderly.

Though detailed statistics are lacking, scattered field reports describe a surge in eye infections, with some health centers in Omdurman locality reportedly managing up to 50 cases daily [8]. The underreporting of keratoconjunctivitis reflects a broader surveillance gap in crisis settings, where non-fatal but debilitating conditions receive lower priority. These serve as micro-indicators of deteriorating environmental health and signal the need for broader syndromic surveillance in crisis contexts.

### Poliomyelitis

Sudan reported new detections of circulating vaccine-derived poliovirus type 2 (cVDPV2) in Port Sudan wastewater samples between September 2023 and January 2024 [9]. This resurgence prompted emergency campaigns targeting over 2.9 million children under five across 51 localities, with additional coverage of 648,300 children in White Nile State.

These events reflect broader regional vulnerabilities, as genomic sequencing has linked Sudanese strains to South Sudan and Chad outbreaks. This transboundary viral movement signals the need for cross-border immunization strategies and coordinated surveillance frameworks.

As of April 2025, the Global Polio Eradication Initiative (GPEI) reports eight WPV1-positive environmental samples in Afghanistan and 25 in Pakistan. Additionally, 15 new cVDPV2 cases have been reported across Chad, Ethiopia, and Niger, with positive ecological samples detected in Chad, Djibouti, Papua New Guinea, and the occupied Palestinian territory [10]. Several countries reporting new polio cases or positive environmental samples are experiencing armed conflict or severe instability. Environmental.

While the rapid response in Sudan is commendable, the reappearance of poliovirus suggests fragility in the routine immunization system. Without sustained surveillance and cross-border coordination, localized outbreaks risk escalating into regional health crises.

### Measles

Measles outbreaks have surged in conflict zones like Darfur and the White Nile. The clustering of measles cases correlates with areas where health worker migration and attacks on facilities have been most severe [11]. The immunization gap isn’t merely a result of health system disruption; it’s also a reflection of fractured governance, cold chain interruptions, vaccine stockouts, and distrust in health services, further exacerbating coverage gaps. The rapid spread of measles underscores the precarious state of disease prevention in the context of a fragmented and overwhelmed healthcare system.

### Dengue fever

Sudan’s dengue fever surge has been particularly severe in Kassala and Khartoum. As of October 28, 2024, 4,544 cases and 12 dengue-related deaths were reported, with Kassala alone accounting for over half of these cases [12]. Traditional mosquito control measures like spraying, larviciding, and waste management have collapsed amid the conflict. The destruction of infrastructure has created ideal breeding grounds for the Aedes mosquito. Additionally, urban destruction, stagnant water accumulation, and disrupted waste disposal systems have transformed dengue from a seasonal to a year-round threat in some regions. The intersection of urban destruction and vector proliferation highlights the changing epidemiological landscape, where traditional control methods are no longer effective.

### Malaria

Malaria cases in Sudan have surged dramatically during the war, with over 3.5 million cases reported in 2024 alone, more than double the number from the previous year [13]. WHO now classifies Sudan among the top five countries globally with the highest malaria burden. This rise is closely tied to conflict-related displacement, environmental disruption from floods, and the collapse of routine prevention programs. Displacement into flood-prone, poorly drained areas and the deterioration of vector control systems have altered the spatial geography of transmission, allowing malaria to thrive even in previously low-endemic zones. Access to antimalarial diagnostics and treatments (like ACTs) remains highly constrained in active conflict areas, where supply chains are severed and health staff are under attack or in hiding.

Moreover, weakened disease surveillance and stock forecasting systems have led to treatment shortages and inconsistent distribution. The current surge reflects not only seasonal or climatic factors, but a broader breakdown of public health governance and protective infrastructure.

Overall, these outbreaks are not isolated epidemiological anomalies amidst the conflict but reflections of deeper systemic collapse. The convergence of war, displacement, disrupted infrastructure, and fragmented public health services has created an ideal environment for infectious disease resurgence. This is not unique to Sudan, similar patterns were seen in Yemen and South Sudan [14, 15], indicating a broader humanitarian-health feedback loop. This loop perpetuates cycles of disease, displacement, and dependency, making it difficult for affected populations to regain health security.

### Challenges to epidemic control in Sudan

The collapsed Sudan’s health infrastructure has created a fragile environment where efforts for epidemic control face considerable challenges. Addressing these barriers requires understanding how war dynamics impact the most basic pillars of outbreak containment.

### Deliberate targeting of health workers

Since the start of the conflict, more than 550 attacks on health infrastructure have been documented, many of which appear to be targeted rather than incidental [3]. Healthcare workers have been assaulted, abducted, or forced to flee, leading to a shortage of critical healthcare professionals from major conflict zones such as Khartoum, El Fasher, and Nyala [1, 4]. With only 20–30% of pre-war health staff remaining in some high-risk states, the human resources necessary for coordinated epidemic response are severely depleted [16].

### Restricted humanitarian access

Humanitarian access remains very difficult. Between September 2024 and March 2025, only 40% of international NGO visa requests were approved, and even those granted access faced routine movement restrictions [1]. Conflict-related road closures, looting, and checkpoint extortion make it nearly impossible to maintain cold chains for vaccines or deliver medical supplies to isolated IDP settlements. Despite over 11 million internally displaced, fewer than 25% of displacement camps have reliable access to primary healthcare services, let al.one outbreak-specific interventions [1, 17].

Moreover, the presence of two rival authorities has produced a chaotic aid landscape. Organizations must navigate dual and often contradictory permit regimes, leading to delays, arbitrary taxation, and denial of access to disease hotspots [1, 2]. In Darfur, for example, multiple cholera alerts could not be verified or contained due to months-long delays in securing travel authorizations for outbreak teams.

### Surveillance system and supply chain collapse

The Integrated Disease Surveillance and Response (IDSR) network has largely disintegrated across RSF-controlled regions, particularly in Darfur, South Kordofan, and parts of Khartoum. Health data reporting is inconsistent or absent, preventing timely outbreak confirmation or contact tracing. Where mobile clinics or INGOs operate, case counts are often incomplete or delayed, undermining real-time response capacity [1, 16]. Mobile internet shutdowns and telecom blackouts further restrict community-based surveillance.

Widespread looting of medical warehouses in Khartoum and Nyala has further disrupted supply chains. Without a central coordination hub and diminished cold chain capacity, even donor-funded vaccine shipments are delayed or rendered unusable upon arrival. Treatment access is similarly constrained; oral rehydration salts, antibiotics, and basic diagnostic kits are unavailable in more than half of IDP sites in the western corridor [16, 17].

### Uncontrolled displacement-driven transmission

Over 11 million internally displaced persons (IDPs) now live in overcrowded shelters, abandoned schools, or makeshift roadside camps, often with no access to safe water or sanitation. In many areas, IDPs are excluded from routine immunization campaigns or early warning systems. These populations are not only vulnerable to epidemics but also act as vectors, particularly as they move between conflict zones and host communities [2]. The emergence of cVDPV2 cases in Sudan and subsequent detections in Egypt, South Sudan, and Yemen reflect this pattern of displacement-driven disease spread across borders [18].

### Displacement and cross-border spillover

The scale and speed of displacement in Sudan since April 2023 have introduced profound risks to national and regional epidemic control. With at least 1.7 million crossing into neighboring countries such as Chad, South Sudan, Egypt, Ethiopia, and the Central African Republic, the country’s conflict now poses a regional health security threat [1, 2].

1. **Displacement as a dual risk factor**.

Internally displaced persons (IDPs) represent both a highly vulnerable group and a potential source of onward disease transmission. Displacement often occurs under extreme duress, with families fleeing without access to water, hygiene, or medical care. In newly formed IDP sites across Sudan, local health systems are overwhelmed, and humanitarian actors report widespread cholera, measles, and conjunctivitis outbreaks in overcrowded shelters [4].

At the same time, displaced populations frequently move between formal camps, informal settlements, and rural host communities, creating a dynamic environment in which pathogens can spread quickly and undetected. These internal movement patterns have contributed to the reemergence of cholera in eight states and measles outbreaks in over 100 localities [16]. The situation is particularly precarious in camps like Zamzam (North Darfur), where WASH infrastructure has collapsed and health access remains limited.

2. **Cross-border health risks**:

Sudan’s porous borders and overlapping displacement passages amplify the risk of international disease spread. Furthermore, thousands of refugees and asylum seekers who originally sought refuge in Sudan now face a reverse displacement to their home countries under duress and without support structures, which significantly raises the risk of cross-border transmission of infectious diseases [4]. 

The risk of cross-border transmission of Polio is increasingly evident, with circulating vaccine-derived poliovirus type 2 (cVDPV2) strains initially detected in Sudan now emerging in neighboring countries like Egypt, South Sudan, and Yemen. This suggests cross-border viral transmission is likely facilitated by refugee movement and breakdowns in vaccination coverage [18]. Similar patterns were observed with cVDPV2 strains originating from the Gaza Strip and later detected in Egypt, underscoring the vulnerability of regional health systems to imported cases.

Cholera, too, poses a significant regional threat. As of early 2025, cholera alerts have been issued in refugee reception areas in eastern Chad, where thousands of Sudanese have settled with limited access to clean water and sanitation [1, 16]. Similar concerns have been raised in South Sudan’s Renk and Malakal counties, which are hosting tens of thousands of arrivals in conditions that can only be described as epidemiologically high-risk [4, 16].

3. **Limited cross-border coordination**:

Despite growing evidence of regional transmission, cross-border epidemic preparedness remains weak. While WHO, UNICEF, and their partners have launched parallel response operations in Sudan and neighboring countries [16], there is no unified regional framework for cross-border disease surveillance, contact tracing, or information sharing. As of early 2025, there are no formal bilateral epidemic response agreements between Sudan’s authorities and its neighbors [1, 4]. This lack of structured coordination jeopardizes containment and regional public health security, particularly in light of rising migration trends and inconsistent vaccination coverage across border zones.

### Recommendations

In light of the protracted conflict and overlapping public health emergencies in Sudan, effective epidemic control will remain limited without addressing the insecurity that directly undermines health service delivery. The following recommendations present a conflict-aware approach to mitigating epidemic risks and rebuilding essential public health functions.

1. Secure humanitarian access and protect health infrastructure.

Efforts to restore healthcare services must be underpinned by secure and sustained humanitarian access. A ceasefire, whether localized or nationwide, is essential to enable the delivery of aid and the reconstruction of health systems. Protection of healthcare facilities and personnel under international humanitarian law must be upheld and monitored by international observers. In the meantime, health response efforts should focus on mobile clinics, field hospitals, and telemedicine where feasible, while long-term plans must be coordinated with conflict resolution efforts and protection guarantees.

2. Support and retain the health workforce under crisis conditions:

Recruiting and keeping healthcare workers is crucial due to the large number of professionals who have been displaced or lost during the conflict. The safety of health workers must be prioritized by establishing safe zones for medical staff and facilities. Where full workforce restoration is unfeasible, task-shifting and decentralized care models involving trained community health workers should be rapidly scaled. Additionally, fast-tracking the training of local healthcare workers, especially in the basics of epidemic response, will help alleviate the personnel shortage. International partners could also send medical teams to assist in areas facing critical shortages. Special attention should be given to providing mental health support for healthcare workers who are managing the burden of conflict and epidemics [19, 20].

3. Rebuild Surveillance Systems with Emergency Functionality:

Disease surveillance must be re-established with an emphasis on flexibility and local adaptability. Conflict-affected regions require simplified, community-based reporting tools that can function without full digital infrastructure. International partners should support early restoration of warning systems and laboratory networks, prioritizing safety, transparency, and decentralization. While regaining control over national reference laboratories is important, this must be approached realistically, with attention to securing high-risk materials and mitigating biosafety threats through technical assistance. The urgent reclamation and fortification of the National Public Health Laboratory in Khartoum should be pursued through negotiated access and emergency international oversight.

4. Expand emergency WASH services and Educational Campaigns:

Preventing cholera and other waterborne diseases requires urgent investment in emergency WASH services for displaced and host communities. Rather than focusing solely on hygiene promotion, responses must address core structural drivers—including the lack of water access points, destruction of sanitation systems, and absence of waste disposal services. WASH interventions must be designed to function independently of centralized infrastructure and should integrate climate- and conflict-adaptive solutions. Flood-prone and overcrowded settings should receive tailored interventions such as raised latrines, safe water trucking, and portable treatment units [21]. Community mobilization should complement, not substitute, service provision.

The prevention of infectious diseases can be promoted through mass media campaigns, community outreach, and school-based programs that aim to educate the public. Educational programs should specifically target displaced populations and rural communities, where access to formal health education is limited. Importantly, messaging must acknowledge real-world constraints, such as the lack of soap, clean water, or privacy. Campaigns should be co-designed and linked to tangible services (e.g., hygiene kits, clean water access). Involving community health workers and trusted local leaders will help foster meaningful engagement.

5. Restore immunization coverage through conflict-resilient strategies:

Immunization programs must be restructured to operate amid ongoing insecurity. This includes deploying outreach teams under humanitarian protection agreements, using short-term ceasefires to access children, and investing in temperature-stable vaccines where cold chain infrastructure is unreliable. Alternative storage methods like solar-powered refrigerators should be explored to support last-mile delivery in off-grid areas. Priority populations include displaced children, communities in active conflict zones, and border regions. Rebuilding national coverage will require a phased, context-aware approach that combines government, NGO, and international coordination.

6. Reassess and prioritize funding for public health emergencies:

The humanitarian funding architecture must respond to the unique public health challenges posed by protracted conflict. This includes dedicated epidemic control funding in the Sudan Humanitarian Needs and Response Plan [17], and prioritization of flexible grants that allow rapid adaptation to changing conditions. Health financing should be better aligned with dynamic field-level epidemiological trends rather than static planning cycles. Capacity-building for local organizations to manage and report on health budgets is also essential to improve accountability and sustainability. Funding should be guided by local epidemiological data and community-identified priorities.

7. Strengthen regional and cross-border health coordination.

Given the regional displacement, epidemic control in Sudan must extend beyond national planning. Establishing cross-border health surveillance, early warning protocols, and vaccine-sharing agreements with neighboring countries, especially Chad, South Sudan, and Ethiopia, is critical to contain outbreaks. Regional health bodies and international organizations should facilitate these collaborations and ensure that refugee and migrant populations are included in national immunization and disease control strategies.

## Data Availability

No datasets were generated or analysed during the current study.

## References

[1] IRC. The Cost of Neglect. Available from: https://www.rescue.org/resource/sudan-cost-neglect. 2025 April. [Cited 2025 May 2].

[2] IOM. Sudan Displacement Tracking Matrix (DTM) Sudan Mobility Update. (16). Available from: https://reliefweb.int/report/sudan/iom-sudan-displacement-tracking-matrix-dtm-sudan-mobility-update-16-publication-date-23-march-2025. 2025 March 23. [Cited 2025 May 2].

[3] Insecurity Insight. Attacks on Health Care in Sudan: Available from: https://insecurityinsight.org. April 2025. [Cited 2025 May 2].

[4] WHO, Sudan Conflict, Emergency C. PHSA. Available from: https://www.who.int/publications/m/item/public-health-situation-analysis--sudan-conflict-(10-march-2025). 2025 March 10. [Cited 2025 May 2].

[5] Associated Press. Cholera kills 58 and sickens about 1,300 others over 3 days in a Sudanese city, health officials say. Available from: https://apnews.com/article/sudan-cholera-outbreak-south-47ac3f39c10eb549785c7fc24608551a. 2025 February 22 [cited 2025 May 20].

[6] WHO. Multi-country cholera outbreak, external situation report. Available from: https://www.who.int/publications/m/item/multi-country-cholera-outbreak--external-situation-report-–24-–20-march–2025 20 March 2025. [Cited 2025 May 20 ].

[7] Malawi Ministry of Health. Malawi Multi-Sectoral Cholera Control Plan (MMCCP) 2025–2030. Available from: https://phim.health.gov.mw/wp-content/uploads/2025/03/Malawi-Multi-sector-Cholera-Control-Plna–1.pdf 2025, [Cited on 2025 May 20].

[8] The World Can’t Wait. Sudan’s Struggle August 11, 2024 – September 1. 2024. Available from: https://worldcantwait.org/2024/09/07/sudans-struggle-august–11–2024-september–1–2024/ 22 September 2024. [Cited 2025 May 20].

[9] UNICEF. Sudan to respond to new emergence of variant poliovirus in Red Sea State. Available from: https://www.unicef.org/sudan/press-releases/sudan-respond-new-emergence-variant-poliovirus-red-sea-state 2024 March 11. [Cited 2025 May 20].

[10] Reliefweb. Polio this week of 16 April 2025. Available from: https://reliefweb.int/report/world/polio-week–16-april–2025 2025 April 17. [Cited 2025 May 20].

[11] Reliefweb.Children at risk of measles outbreak in Rokero, Sudan. Available from: https://reliefweb.int/report/sudan/children-risk-measles-outbreak-rokero-sudan 2024 December 19. [Cited 2025 May 20].

[12] UN News. First famine, now cholera and dengue fever surge hits war-torn Sudan. Available from: https://news.un.org/en/story/2024/11/1156461#:~:text=to%20be%20attacked.-,First%20famine%2C%20now%20cholera%20and%20dengue,surge%20hits%20war%2Dtorn%20Sudan&text = War%20in%20Sudan%20has%20devastated,threshold%E2%80%9D%2 C%20humanitarians%20have%20warned. 2024 November 4. [Cited 2025 May 20 ].

[13] DABANGA. World Malaria Day. WHO stats show massive surge amid war in Sudan. Available from: https://www.dabangasudan.org/en/all-news/article/world-malaria-day-who-stats-show-massive-surge-amid-war-in-sudan 2025 April 25. [Cited 2025 May 20].

[14] Reliefweb Y. Violence Against Health Care in Conflict 2023, Available from: https://reliefweb.int/report/yemen/yemen-violence-against-health-care-conflict–2023-enar#:~:text=Attacks%20on%20health%20care,%20combined,functional%20due%20to%20insufficient%20resources. 2024 September 24. [Cited 2025 May 20].

[15] ICRC. South Sudan: human toll of conflict and violence strains healthcare system. Available from: https://www.icrc.org/en/document/south-sudan-human-toll-conflict-and-violence-strains-healthcare-system 2023 February 2. [Cited 2025 Ma 20].

[16] WHO Health Cluster Sudan Bulletin. Available from: https://reliefweb.int/report/sudan/health-cluster-sudan-bulletin-january–2025. 2025 April 29. [Cited 2025 May 4].

[17] OCHA.Sudan.Humanitarian Access Snapshot. Available from: https://reliefweb.int/report/sudan/sudan-humanitarian-access-snapshot-march-2025. 2025 April 5 [Cited 2025 May 4].

[18] Global Polio Eradication Initiative. Outbreak countries.Available from: https://polioeradication.org/news-categories/outbreak-countries/[Cited 2025 May 4].

[19] World Health Organization and Federal Ministry of Health. Sudan Outbreaks Dashboard. Available from: https://worldhealthorg.shinyapps.io/OutbreaksDashboard/. [Cited: 2025 May 20].

[20] President ICRCICRC. The obstacles that block humanitarian access must be immediately overcome. Available from: https://www.icrc.org/en/where-we-work/sudan 2024 April 15 [cited 2025 May 20].

[21] CARE International. Sudan: 500 Days of Death, Devastation and Destruction. Available from: https://www.care.org/news-and-stories/press-releases/sudan-500-days-of-death-devastation-and-destruction/. 2024 August 27 [cited 2025 May 20].

